# Cross-Species Susceptibility of Emerging Variants of SARS-CoV-2 Spike

**DOI:** 10.3390/genes15101321

**Published:** 2024-10-14

**Authors:** Meng Li, Fei Lv, Zihao Li, Chenyu Zhao, Xiao Wang, Pingfen Zhu, Xuming Zhou

**Affiliations:** 1 Key Laboratory of Animal Ecology and Conservation Biology, Institute of Zoology, Chinese Academy of Sciences, Beijing 100101, China; limeng@ioz.ac.cn (M.L.); lvfe1024@163.com (F.L.); lzh_starrk@163.com (Z.L.); zhaochenyu22@ioz.ac.cn (C.Z.); wxiao@ioz.ac.cn (X.W.); zhupingfen@ioz.ac.cn (P.Z.); 2 University of Chinese Academy of Sciences, Beijing 100049, China

**Keywords:** SARS-CoV-2, spike protein mutations, cross-species transmission, viral infectivity

## Abstract

Background: The continuous evolution of SARS-CoV-2 and the emergence of novel variants with numerous mutations have heightened concerns surrounding the possibility of cross-species transmission and the establishment of natural animal reservoirs for the virus, but the host range of emerging SARS-CoV-2 variants has not been fully explored yet. Methods: We employed an in vitro model comprising VSV∆G* pseudotyped viruses bearing SARS-CoV-2 spike proteins to explore the plausible host range of SARS-CoV-2 emerging variants. Results: The overall host tropism of emerging SARS-CoV-2 variants are consistent with that of the SARS-CoV-2 wuhan-hu-1 strain with minor difference. Pseudotyped viruses bearing spike protein from RaTG13 and RmYN02 can enter cell cultures from a broad range of mammalian species, revealing that mink and hamsters may act as potential intermediate hosts. We further investigated 95 potential site-specific mutations in the SARS-CoV-2 spike protein that could impact viral infectivity across different species. The results showed that 13 of these mutations notably increased the transduction rates by more than two-fold when compared to the wild-type spike protein. Further examination of these 13 mutations within cell cultures from 31 different species revealed heightened sensitivity in cells derived from palm civets, minks, and Chinese horseshoe bats to the VSV∆G*-SARS2-S mutants. Specific mutations, such as L24F, R158G, and L212I, were seen to significantly enhance the capacity for SARS-CoV-2 of cross-species transmission. Conclusions: This study offers critical insights for the ongoing surveillance and monitoring efforts of SARS-CoV-2 evolution, emphasizing the need for the vigilant monitoring of specific mutations in both human and animal populations.

## 1. Introduction

The ongoing COVID-19 pandemic, caused by the highly contagious SARS-CoV-2 virus, remains a major concern for public health, global economy, and societal stability. As of 13 December 2023, the worldwide burden of COVID-19 has surpassed 772 million confirmed cases and resulted in more than 6.9 million deaths, as reported by the World Health Organization. Despite some alleviation in the COVID-19 pandemic situation, SARS-CoV-2 is undergoing rapid evolution with the frequent emergence of novel mutations during this ongoing crisis [1].

At the beginning of the pandemic, notable instances of widespread animal infection were reported on mink farms, which provided evidence of two-directional transmission between humans and animals [2]. Fortunately, most instances of human-to-animal transmission of SARS-CoV-2 are sporadic with limited animal-to-animal transmission. However, in 2021, multiple studies reported spillover events from humans to free-ranging white-tailed deer followed by subsequent deer-to-deer transmission [3,4,5]. Importantly, there have also been indications of potential spillback from various animals, including hamsters, mink, cats, and white-tailed deer, to humans [2,5]. Up to 20 September 2024, at least 928 outbreaks of SARS-CoV-2 had been recorded, affecting 34 different animal species worldwide [6]. The extensive spread and reciprocal nature of SARS-CoV-2 transmission amplify concerns regarding the emergence of variants with mutations that could enable the virus to establish a natural animal reservoir [7,8]. We had tested the possible host tropism of the SARS-CoV-2 wuhan-hu-1 strain. However, the host range for SARS-CoV-2 novel variants, especially for variants of concerned (VOC) and variants of interests (VOI), are rarely tested.

The spike protein of SARS-CoV-2, along with its interaction with the ACE2 receptor (S-ACE2 interaction), plays a crucial role in the virus’s entry into cells and its capacity to transmit across species [9,10]. A multitude of mutations within the spike protein significantly enhance the spillover potential of SARS-CoV-2 [11,12]. For instance, substitutions N439K, N501Y, S477N, D614G, and H655Y in the S proteins enhance pathogenicity and transmission among humans [13,14]. On the other hand, substitutions N501Y, K417N, E484K, Q493H/K, and Q498H are posited to be pivotal for the virus’s adaptation to rodents [12,15]. Furthermore, the mutations D614G and K417N lead to a marked increase in the binding affinity between the spike protein and ACE2 receptors from a wide range of animal species, including but not limited to mice, pangolins, bats, minks, ferrets, cats, dogs, sheep, and rabbits [16]. Interestingly, while wild-type mice do not succumb to infection with the authentic Wuhan strain of SARS-CoV-2, SARS-CoV-2 Omicron variants carrying N501Y mutation have demonstrated an enhanced ability to infect wildtype murine, which holds additional implications for human exposure risk [17]. In our early study, we investigated the impact of all S protein high-frequency mutations on alterations in host tropism for SARS-CoV-2, and found that specific mutations in the S protein (i.e., del69-70, D80Y, S98F, T572I, and Q675H) significantly alter its host tropism [18].

Nonetheless, novel variants of SARS-CoV-2, characterized by unpredictable mutations, continue to emerge. This raises concerns regarding the spillover of SARS-CoV-2 to animals and the establishment of a new natural animal reservoir, thereby posing an even more challenging threat to public health [8]. In particular, studies have shown the sustained and accelerated evolution of SARS-CoV-2 within various animal hosts, including domestic animals like cats and dogs, as well as wildlife such as ferrets and white-tailed deer [19,20]. Thus, examining the host tropism of emerging variants, as well as potential mutations in the spike protein, is critical for informing effective prevention and control strategies in the evolving landscape of the pandemic.

To address this need, we utilized an in-vitro infection model consisting of (i) cell cultures derived from 49 mammalian species belonging to 11 orders and (ii) green fluorescent protein (GFP)-encoding vesicular stomatitis virus (VSV) bearing the S protein from both SARS-CoV-2 and SARSr-CoVs. Through this model, we assessed the spike protein-mediated cellular entry of seven variants of SARS-CoV-2, two SARSr-CoVs, and 95 potential site-specific mutations that have yet to occur. Additionally, we focused on 13 specific mutations with a likelihood of emerging in human populations and examined their effects on viral entry into various animal cell cultures. Our findings demonstrated that the host tropisms of emerging variants of SARS-CoV-2 are largely similar with minor changes, while mutations L24C, R158N, and L212Y possess the potential to enhance cross-species transmission capabilities of SARS-CoV-2. These insights are crucial for ongoing surveillance and monitoring efforts regarding the evolution of SARS-CoV-2.

## 2. Material and Methods

### 2.1. Ethic Statement

The experiments were carried out in a Biosafety Level 2 (BSL-2) facility and approval was obtained from the Animal Ethics Committee of the Institute of Zoology, Chinese Academy of Sciences (permission no: IOZ-IACUC-2021-163).

### 2.2. Cell Culture Models

The primary cell cultures utilized in this study (Supplementary Table S1) were obtained from companion animals, farm animals, and wildlife species following previously established protocols [21]. To minimize the impact of variables such as sex, age, immunity, and other related factors, we sampled healthy young adult males from each species whenever possible. The animals were euthanized using CO_2_ or pentobarbital calcium (200 mg/kg). The carcasses were dissected under aseptic conditions to collect kidneys, lungs, hearts, or other tissues. In order to prevent tissue dehydration, all the specimens were immersed in sterile PBS supplemented with a 2% Penicillin–Streptomycin solution (Gibco, Grand Island, NY, USA) until cellular extraction. Each tissue sample was then transferred into a 35 mm dish and finely minced using dissecting scissors. The resulting tissue fragments were subsequently placed into a 50 mL conical tube for enzymatic digestion utilizing 0.25% EDTA-trypsin (Gibco, New York, NY, USA) at 37 °C for a duration of 30 min. During the digestion, the mixture was shaken vigorously every 10 min. The trypsin digestion was stopped by adding FBS into the conical tube followed by pipetting up and down for 3 min to break clumps of tissue fragments. The resulting solution was centrifuged at 250 g for 5 min at 4 °C to collect pellet cells, which were then resuspended in DMEM-F12 medium (Gibco, Grand Island, NY, USA) containing 10% FBS (Penrose, Auckland, New Zealand) and seeded into dishes of appropriate size. All the dishes were incubated at standard conditions of temperature (37 °C) and CO_2_ concentration (5%). Daily monitoring ensured proper cell growth without contamination while regular passages occurred upon reaching confluence. All the primary cells isolated and cultured in DMEM-F12 medium contained additional supplements, including a Penicillin–Streptomycin solution (1%) along with HEPES buffer solution (20 mM).

The following cell lines were cultured in DMEM: Huh-7 (Homo sapiens, liver), HEK-293T (Homo sapiens, embryonic kidney), Vero (*Cercopithecus aethiops*, kidney), OK (*Monodelphis domestica*, kidney), BHK-21 (*Mesocricetus auratus*, kidney), MDBK (*Bos taurus*, kidney), PK-15 (*Sus scrofa*, kidney), F81 (*Felis catus*, kidney), and Mv.1.lu (*Neovison vison*, lung). All these cell lines were incubated at 37 °C with 5% CO_2_ using specified culture mediums supplemented with 10% fetal bovine serum (FBS, Gibco, USA), a 1% Penicillin–Streptomycin solution, and 20 mM Hydroxyethylpiperazine Ethane Sulfonic Acid (HEPES, Gibco, Paisley, UK).

### 2.3. Prediction of Potential Mutations

First, we hypothesized that spike proteins have a tolerance to mutations at high-frequency mutation sites [22]. Then, we selected high-frequent mutations with a frequency of >0.003; within the RBD regions, we selected high-frequent mutations with a frequency of >0.001 (CoV-GLUE) [23]. Then, we predicted the mutations that potentially emerged in these sites based on the principle of codon degeneracy. In detail, we changed the number 1 and number 2 nucleotide randomly, while keeping the number 3 nucleotide unchanged. Then, we compared the potential mutations to currently known mutations, and removed the mutations that had been observed previously. In total, 191 potential mutations were observed, and 95 of them have never been recorded. The expression of the spike protein may be affected by a mutated codon resulting from codon bias. Therefore, the codon encoding the target amino acid was substituted with a codon-optimized version to minimize the impact of codon bias.

### 2.4. Plasmids and Site-Directed Mutagenesis

Human codon-optimized S genes of SARS-CoV-2 Wuhan-Hu-1 strain (GenBank accession No. NC_045512.2), BatCoV-RaTG13 (GISAID accession No. EPI_ISL_402131), BatCoV-RmYN02 (GISAID accession No. EPI_ISL_412977) were synthesized and cloned into pcDNA3.1 vector for the pseudotyped viruses’ generation. S genes of SARS-CoV-2 B.1.617.2 (Delta), XBB (Omicron), XBB.1.5 (Omicron), XBB.1.16 (Omicron), EG.5.1 (Omicron), BA.2.75 (Omicron), and BA.2.86 (Omicron) were purchased from the Addgene plasmid repository. Site-directed mutagenesis was performed using pcDNA3.1-SARS2-wuhan-hu-1 as a template. We designed forward primers with a melting temperature (Tm) of approximately 75 °C and placed the mutations in the middle, while corresponding reverse complementary sequences were selected as reverse primers. A 20 μL PCR mix was prepared, containing 10 μL of 2× Phanta Max Master Mix (Vazyme, Nanjing, China), 10 pmol of forward and reverse primers, and 10 ng of template plasmid. After performing 20 cycles of site-directed mutagenesis PCR, we digested the template plasmid using DpnI restriction endonuclease (NEB, Ipswich, MA, USA). Then, we transformed the PCR product into *E. coli* DH5a competent cells (Transgen, Beijing, China) and selected single clones for sequencing. All the plasmids were extracted using a QIAGEN Plasmid Maxi Kit (Qiagen, Germantown, MD, USA). To prevent contamination during cell culture when generating pseudotyped viruses, we inactivated the extracted plasmids in a water bath at 65 °C for 30 min. The concentrations of the plasmids were quantified by Nanodrop2000 (Thermo Scientific, Wilmington, DE, USA).

### 2.5. WB Analysis of the Expression of S Proteins

To ensure the correct and abundant expression of S proteins derived from SARS-CoV-2 wuhan-hu-1, emerging variants, mutated S proteins, as well as SARSr-CoVs in HEK-293T cells, a Western Blot (WB) analysis was employed for protein detection. Specifically, HEK-293FT cells were cultured in 6-well plates one day prior to transfection. When the HEK-293T cells reached 80–90% confluency, each plasmid (3 μg) was transfected into the cells using Lipofectamine 3000 according to the manufacturer’s instructions (Thermo Fisher Scientific, Waltham, MA, USA). After 48 h post-transfection, the supernatants were discarded and the cells were washed twice with PBS. Subsequently, the cells were directly treated with 300 μL of 2× SDS loading buffer. The lysates were collected into cold 1.5 mL Eppendorf tubes and subjected to ultrasonic treatment. A total of 5 μL from each sample was utilized for WB analysis. The SDS-PAGE electrophoresis and transmembrane were performed as regular procedure. SARS-CoV/SARS-CoV-2 (COVID-19) spike antibody (Genetex, Irvine, CA, USA), β-Actin Mouse mAb (ABclonal, Wuhan, China) and HRP-conjugated Goat anti-Mouse IgG (H+L) (ABclonal, Wuhan, China) were used for S protein and β-actin detection.

### 2.6. Pseudotyped Virus Package and Quantification

We constructed pseudotyped viruses incorporated with S proteins from SARS-CoV-2 using a protocol reported in previous research [24]. HEK-293T cells that reached 70% to 90% confluence were transfected with 15 μg of plasmids encoding SARS-CoV-2 spike proteins using Lipofectamine 3000. At the same time, plasmids encoding VSV-G proteins were either transfected or mock-transfected into the HEK-293T cells as positive and negative controls. The transfected cells were cultured at 37 °C with 5% CO_2_. 24 h later, we infected the cells with VSVΔG*-G viruses at a multiplicity of infection (MOI) of 3 to 4 and let them incubate for two hours. Following this step, we gently discarded the cell supernatant and rinsed the cells twice with warm PBS. Next, we added 10 mL of fresh DMEM enriched with 10% FBS, a solution containing Penicillin–Streptomycin at a concentration of 1%, and HEPES at a concentration of 20 mM to each dish before placing them back in the incubator at 37 °C with an atmosphere rich in CO_2_. After another day, we checked these cells for EGFP positivity; afterwards, we collected the supernatant fluid, centrifuged it, filtered it out, and divided it into aliquots. The pseudotyped viruses bearing novel SARS-CoV-2 S proteins were named VSVΔG*-SARS2, VSVΔG*-SARS2-B.1.617.2, VSVΔG*-SARS2-XBB, VSVΔG*-SARS2-XBB.1.5, VSVΔG*-SARS2-XBB.1.16, VSVΔG*-SARS2-EG.5.1, VSVΔG*-SARS2-BA.2.75, VSVΔG*-SARS2-BA.2.86, VSVΔG*-RaTG13, and VSVΔG*-RmYN02. The library of pseudotyped viruses bearing mutated SARS-CoV-2 S proteins were named VSVΔG*-SARS2mut. All of the pseudotyped viruses were stored at −80 °C. Repeated freezing–thawing cycles were avoided.

Due to the variation in receptor usage among different S proteins, quantifying the titer of VSV pseudotyped viruses through the infection of HEK-293T-hACE2 cells has proven challenging. To address this issue, we devised a relative quantification strategy utilizing H1N1 influenza virus as a reference. Specifically, 100 μL of each VSV pseudotyped virus was combined with 10 μL of H1N1 influenza virus. Subsequently, total RNAs were extracted using TRIZol regent following the manufacturer’s instructions. The abundance of the VSV pseudotyped viruses was determined by qPCR with the influenza M gene serving as a reference gene. Finally, the infection dosage of the VSV pseudotyped viruses was adjusted to VSVΔG*-SARS2 based on the results obtained from relative quantification. The SYBR Green qPCR primers used in these experiments were listed as follows:

H1N1_M_For: 5′-ATTTGCCTATGAGACCGATGCT-3′;

H1N1_M_Rev: 5′-AGGATGGGGGCTGTGACC-3′ [25];

VSV_N_For: 5′-CGGAGGATTGACGACTAATGC-3′;

VSV_N_Rev: 5′-ACCATCCGAGCCATTCGA-3′ [26].

The standard cycling conditions were 95 °C for 2 min, followed by 40 cycles of 95 °C for 30 s, 60 °C for 30 s, and 72 °C for 30 s, followed by a melting curve analysis.

### 2.7. Infection of Cell Cultures by VSV Pseudotyped Viruses

Cell cultures were seeded into 48-well plates (10,000 cells per well), cultured in DMEM-F12 supplemented with 10% FBS, a 1% Penicillin–Streptomycin solution, and 20 mM HEPES. Upon reaching 70% confluence, the culture supernatants were discarded. Subsequently, the cells were rinsed twice using warm PBS buffer supplemented with a 1% Penicillin–Streptomycin solution. Following this step, the cell cultures were infected with pseudotyped viruses, including those bearing wild-type and mutated S proteins, VSV-G glycoproteins, as well as a negative control (culture supernatant harvested from mock transfected cells). Each infection experiment was performed in triplicate independently. For VSVΔG*-SARS2 infection analysis, an MOI of 0.15 was utilized to infect the Huh-7 cell line, and the dosage of VSVΔG*-SARS2-B.1.617.2, VSVΔG*-SARS2-XBB, VSVΔG*-SARS2-XBB.1.5, VSVΔG*-SARS2-XBB.1.16, VSVΔG*-SARS2-EG.5.1, VSVΔG*-SARS2-BA.2.75, and VSVΔG*-SARS2-BA.2.86 were adjusted to match that of VSVΔG*-SARS2 based on qPCR analysis results. The Huh-7 cell line was infected with VSVΔG*-RaTG13 at an MOI of 0.15 for infection analysis, and the dosage of VSVΔG*-RmYN02 was adjusted to match that of VSVΔG*-RaTG13 based on the qPCR analysis results. 50 μL of VSVΔG*-noG was used to infect each cell culture. The viruses were maintained in the culture medium supplemented with 1% FBS and a 1% Penicillin–Streptomycin solution for 24 h before undergoing microscope observation and flow cytometry analysis to identify GFP-positive cells.

### 2.8. Flow Cytometry Analysis

Cells infected or mock-infected with pseudotyped viruses were observed using a fluorescent microscope. After discarding the culture medium, cells were rinsed twice in warm PBS and then subjected to 0.25% EDTA-trypsin digestion for 5 min. Subsequently, the cells were collected into 1.5 mL tubes and washed twice with PBS to remove trypsin. To eliminate cell clumps, the cells were filtered through a 70 μm cell strainer (Corning, Tewksbury, MA, USA). The final cell suspension was kept on ice in a dark chamber. Flow cytometry analyses were performed using CytoFLEX (Beckman Coulter, Indianapolis, IN, USA). Cells infected with VSVΔG*-G viruses or negative control samples were utilized to optimize the voltage settings for FSC, SSC, and FITC detectors, as well as quantify the populations of positive or negative particles. For each infection assay, at least 5000 total cells were analyzed. The transduction rates are presented as mean ± SD based on three independent replicates.

### 2.9. Molecular Dynamic Simulations and Interaction Prediction

The interface between the S proteins of SARS-CoV-2 and human ACE2 were examined by molecular dynamic (MD) simulations using Amber 20 [27,28,29]. We first downloaded the spike-ACE2 crystal structure (PDB ID: 7A94) from the Protein Data Bank [9]. The 3D crystal structure served as a template to prepare 3D models for SARS-CoV-2 S mutates and hACE2 using PyMol software (Version 2.5.0). Next, each system was evaluated using tleap and placed in a cubic periodic box of TIP3P water, extending 10 Å from the solute. We then neutralized the system with an appropriate number of counter ions, either Na+ or Cl−, followed by parameterization with the Amber ff14SB force field [30]. Next, 10,000 steps of energy minimization, including 5000 steps of the steepest descent method and 5000 steps of the conjugate gradient minimization were performed. Each system was then heated to 300 K in the NVT ensemble by 0.2 ns. The minimization, heating, and equilibrium simulations were performed with strong constraints (500 kcal/mol/Å2) on heavy atoms with the sander program in Amber20. A total of 30 ns MD simulations were performed at a constant temperature of 300 K using the NPT ensemble and pmemd.cuda. The system temperature was kept stable using Langevin dynamics. A cutoff distance of 10 Å was set to distinguish between the Van der Waals interactions and short-range electrostatic energies. We employed the particle mesh Ewald (PME) method to calculate long-range electrostatic interactions. From the equilibrium trajectory, we extracted at least 3000 snapshots to obtain the final average structure for each RBD-ACE2 complex. The MM/GBSA method helped us to determine the binding free energy (ΔG), which we then decomposed into contributions from each individual residue [31].

### 2.10. Statistical Analysis

We used R for statistical analysis and plotting the data. All the histogram values were expressed as mean ± SD. When the collected cell cultures from the different species were infected by the VSVΔG*-SARS2 bearing S protein from the different variants, we classified the transduction rates into four categories (i.e., minimal, slight, moderate, and efficient transduction) based on the following principles. First, we compiled the quantiles of all the transduction rates and calculated the interquartile range (IQR) based on these values. Subsequently, we determined the standard deviation (SD) of the transduction rates from minimum to Q1, which we designated as SD_Q1_. If a cell culture exhibited a transduction rate of less than ten times SD_Q1_, it was categorized as a negligible infection; those with transduction rates between 10 times SD_Q1_ and Q3 were classified as slight infections; rates ranging from Q3 to Q3 + 1.5 * IQR were considered moderate infections, while those exceeding Q3 + 1.5 * IQR were classified as efficient infections. A two-sided Wilcoxon test was adopted to compare the capacity of different VSVΔG* pseudotyped viruses to transduce different cell cultures compared to VSVΔG*-SARS2.

## 3. Results

### 3.1. Comparative Susceptible Analysis of Novel SARS-CoV-2 Variants to Multiple Animals

**Figure 1:**
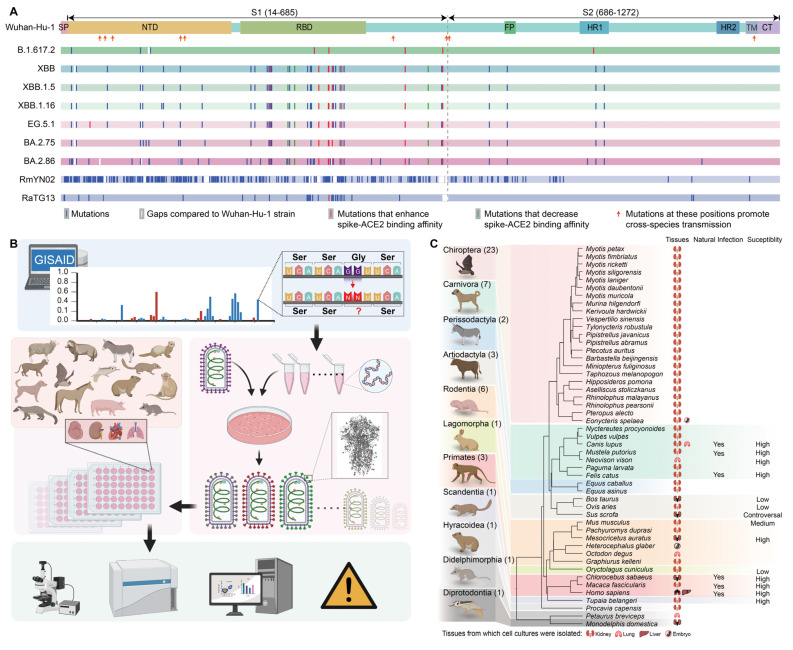
Schematic description of in vitro infection model. (**A**) A comparative analysis of S proteins from SARS-CoV-2 wuhan-hu-1, B.1.617.2 (Delta), XBB, XBB.1.5, XBB.1.16, EG.5.1, BA.2.75, BA.2.86, Bat-CoV RaTG13, and Bat-CoV RmYN02. The short lines with different colors represent amino acid variations: the blue lines represent amino acid substitutions; the white lines (or fragments) represent deletions or gaps; the red or green lines represent one of the substitutions at this position that enhances or decreases the receptor binding affinity. The orange arrows mean mutations at these sites enhance the capacity for cross-species transmission. (**B**) A schematic description of the in vitro infection model that comprised GFP-encoding VSV pseudotyped viruses and cell cultures derived from multiple animals. (**C**) The tested species were displayed as their position in the phylogenetic tree (TimeTree, https://timetree.org/home (accessed on 9 October 2024)). Primary cell cultures, immortalized cells, and cell lines derived from forty-nine species were tested in this research. while the susceptibility towards SARS-CoV-2 infection was experimentally or naturally tested in 14 different species. High represents the species that are highly susceptible to SARS-CoV-2 infection; medium represents the species that can be occasionally infected by SARS-CoV-2; low represents the species that are rarely infected by SARS-CoV-2.

Along with the circulation of SARS-CoV-2, thousands of novel variants emerged frequently. Some species, which were resistant to SARS-CoV-2 wuhan-hu-1 strains, were susceptible to novel SARS-CoV-2 variants [32]; the risk of SARS-CoV-2 establishing a natural reservoir host should be carefully assessed. We analyzed of the spike protein in various SARS-CoV-2 variants, including wuhan-hu-1, B.1.617.2 (Delta), XBB (omicron), XBB.1.5 (omicron), XBB.1.16 (omicron), EG.5.1 (omicron), BA.2.75 (omicron), and BA.2.86 (omicron). Compared to the wuhan-hu-1 strains, these variants exhibited amino acid substitutions or deletions ranging from 10 to 63 with amino acid sequence identities ranging from 99.2% to 94.5% (Supplementary Table S2). Some of these substitutions or indels were shown to enhance or decrease spike-hACE2 binding affinity (Figure 1A). In our previous research, we tested the comparative susceptibility of the SARS-CoV-2 wuhan-hu-1 strain on a total of 55 mammalian species and found five mutations outside RBD which conferred SARS-CoV-2 with specific animal infectivity ability: del69 -70, D80Y, S98F, T572I, and Q675H; fortunately, none of these mutations were observed in any of the tested variants (Figure 1A).

To explore the plausible host range of these SARS-CoV-2 variants, we generated eight GFP-expressing pseudotyped viruses carrying relevant S proteins, namely VSVΔG*-SARS2, VSVΔG*-SARS2-B.1.617.2, VSVΔG*-SARS2-XBB, VSVΔG*-SARS2-XBB.1.5, VSVΔG*-SARS2-XBB.1.16, VSVΔG*-SARS2-EG.5.1, VSVΔG*-SARS2-BA.2.75, and VSVΔG*-SARS2-BA.2.86, respectively. Subsequently, these pseudotyped viruses were employed to infect 48 cell cultures derived from 44 species belonging to 10 orders, including Didelphimorphia (one species), Diprotodontia (one species), Scandentia (one species), Primates (three species), Lagomorpha (one species), Rodentia (six species), Artiodactyla (two species), Perissodactyla (two species), Carnivora (seven species), and Chiroptera (twenty species) (Supplementary Table S3). The 48 cell cultures comprised of 38 primary cell cultures, one immortalized cell culture, and 10 established cell lines. Among these, forty out of the 48 cell cultures were derived from kidney tissues exhibiting abundant expressions of ACE2s.

**Figure 2:**
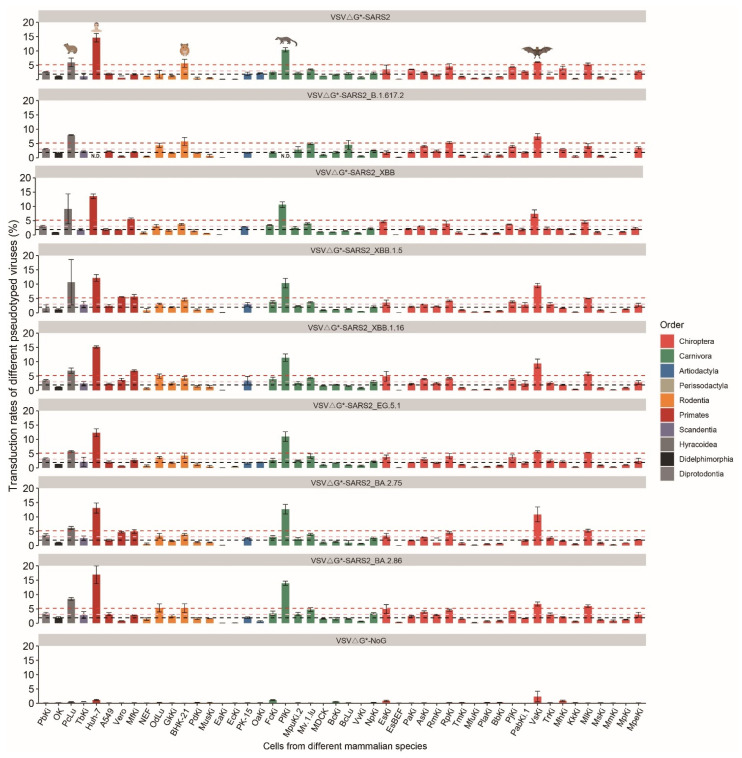
VSV pseudotyped viruses bearing S proteins from different SARS-CoV-2 variants can transduce cell cultures from a broad range of mammalian species. The transduction rates (mean ± SD, n = 3) of cell cultures from human beings, hamsters, palm civets, Rock hyrax, and Asian particolored bat can be efficiently transduced by constructed SARS-CoV-2 pseudotyped viruses. The bars under the black lines represent that these cell cultures were minimally transduced. The pink and red dash lines represent the standard for defining moderate and efficient transduction, respectively.

Consistent with our previous study, we observed that all eight constructed VSV pseudotyped viruses exhibited the ability to efficiently transduce diverse mammalian cell cultures (Figure 2). VSVΔG*-SARS2-B.1.617.2, VSVΔG*-SARS2-XBB, VSVΔG*-SARS2-XBB.1.5, VSVΔG*-SARS2-EG.5.1, and VSVΔG*-SARS2-BA.2.75 displayed no significant differences in their overall transduction rates compared to VSVΔG*-SARS2, and higher transduction rates were observed in VSVΔG*-SARS2-XBB.1.16- (*p* < 0.01) and VSVΔG*-SARS2-BA.2.86- (*p* < 0.001) infected cell cultures (Supplementary Figure S1A). In addition to the human (Huh-7) cell line, rock hyrax (PcLu), palm civet (PlKi), Chinese water myotis (MlKi), and Asian particolored bat (VsKi) cell cultures were effectively transduced by all eight pseudotyped viruses, suggesting that these species could potentially serve as reservoir hosts for SARS-CoV-2 variants (Figure 2). The tested VSV pseudotyped viruses displayed no difference in transduce Human Huh-7 cell line (Supplementary Figure S1B). But, we noted that VSVΔG*-SARS2-B.1.617.2, VSVΔG*-SARS2-XBB, VSVΔG*-SARS2-XBB.1.5, VSVΔG*-SARS2-XBB.1.16, and VSVΔG*-SARS2-BA.2.86 have higher capacities to transduce Asian particolored bat cell cultures (VsKi) (Supplementary Figure S1C). We also noted that VSVΔG*-SARS2-B.1.617.2 had a higher capacity to transduce dog cell cultures (BcLu), while the other SARS-CoV-2 variants displayed decreased efficiencies in transducing BcLu (Supplementary Figure S1D). These results suggested that the overall host range of SARS-CoV-2 are consistent with minor changes, and that we should pay continuous attention to the monitoring of SARS-CoV-2 in companion animals, farm animals, and wild animals.

### 3.2. Comparative Susceptible Analysis of RaTG13 and RmYN02 on Multiple Animals

Bat-CoV RaTG13 and Bat-CoV RmYN02 are SARS-related coronaviruses (SARSr-CoV), and are phylogenetically associated with SARS-CoV-2. The spike protein of RaTG13 and RmYN02 have 33 and 344 substitutions or indels compared to SARS-CoV-2 wuhan-hu-1 strains, with sequence identities of 97.3% and 72.4%, respectively (Supplementary Table S2). Interestingly, the number of amino acid substitutions in RaTG13 is less than that in the SARS-CoV-2 XBB, XBB.1.5, XBB.1.16, EG.5.1, BA.2.75, and BA.2.86 variants (Supplementary Table S2). Previous studies showed that RaTG13 has the capacity to utilize the human ACE2 (hACE2) receptor, indicating that RaTG13 might spillover to human society directly or as an intermediate host [33]. Exploring the possible host range of RaTG13 and RmYN02 helps to predict the possible intermediate hosts that transmit viruses to humans. Therefore, we constructed a VSVΔG* pseudotyped virus bearing S proteins from RaTG13 and RmYN02, which were named as VSVΔG*-RaTG13 and VSVΔG*-RmYN02, respectively.

**Figure 3:**
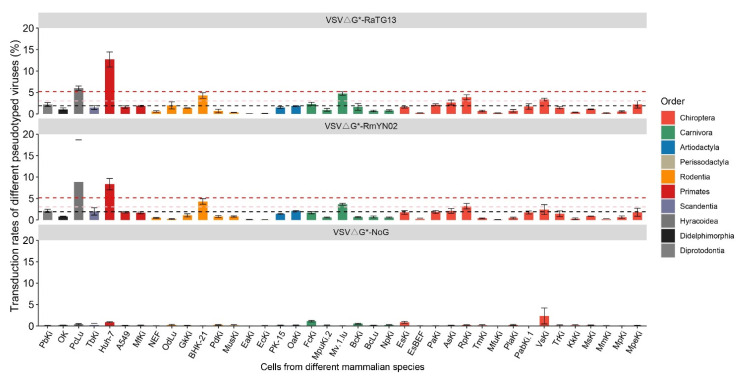
VSV pseudotyped viruses bearing S proteins from Bat-CoV RaTG13 and Bat-CoV RmYN02 can transduce cell cultures from a broad range of mammalian species. The pink and red dash lines represent the standard for defining moderate and efficient transduction, respectively. The bar was presented as mean ± SD (n = 3).

VSVΔG*-RaTG13 and VSVΔG*-RmYN02 infected 40 cell cultures. Based on our results, we found that VSVΔG*-RaTG13 and VSVΔG*-RmYN02 had the capacity to transduce cell cultures from a broad range of mammalian species (Figure 3). When the cell cultures were infected by the same dosage of pseudotyped viruses, VSVΔG*-RaTG13 had higher transduction efficiencies than VSVΔG*-RmYN02 (Figure 3, Supplementary Figure S2A). We further focused on cell cultures from bat species; the results showed that VSVΔG*-RaTG13 displayed a higher capacity to transduce bat cells than VSVΔG*-RmYN02 (Supplementary Figure S2B). Pet animals and domestic animals, such as cats, dogs, horses, pigs, hamsters, and common degu, can make contact with human beings at high frequencies; we found that cell cultures FcKi (2.33%), BcKi (1.64%), EaKi (0.04%), PK-15 (1.51%), BHK-21 (4.30%), and OdLu (1.97%) from these species are slightly or moderately infected by VSVΔG*-RaTG13 and VSVΔG*-RmYN02 (Figure 3). Six cell cultures from carnivora animals were tested; among these species, the cell line from mink (Mv.1.Lu, 4.76%) displayed the highest transduction rates (Figure 3, Supplementary Table S2). The overall results showed that Bat-CoV RaTG13 and Bat-CoV RmYN02 can be transmitted to humans via several hosts, especially by minks.

### 3.3. Infection of Human Cells by Pseudotyped Viruses Bearing Mutated S Proteins

**Figure 4:**
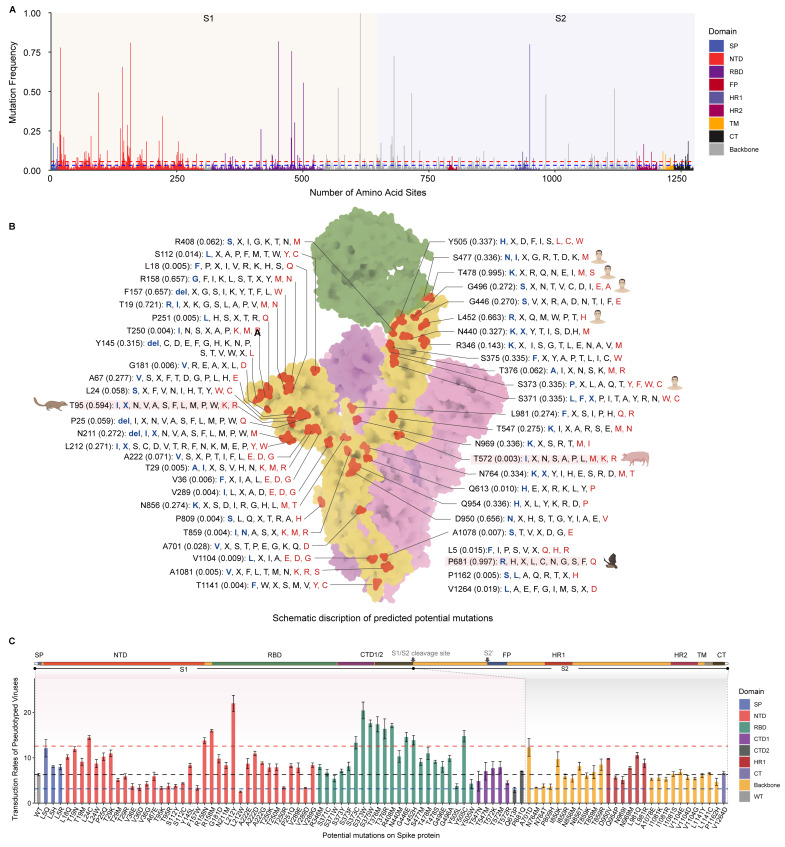
The prediction and functional analysis of potential mutations in SARS-CoV-2 wuhan-hu-1 spike protein. (**A**) The statistics of the mutation rates of each site. The red line means the threshold for mutation rate equals 0.3%; the blue line means the threshold for mutation rate equals 0.1%. (**B**) 52 sites were chosen based on the mutation rates of each site of spike protein. The most frequent mutation is marked in blue; other mutations are marked in black, while potential mutation are marked in red. Among these sites, mutations at 373, 446, 452, 477, 478, and 496 enhanced the viral entry of SARS-CoV-2. The mutations at 95, 572, and 681 promote spike mediate viral entry of SARS-CoV-2 into primary cells derived from specific wild animals. These mutations are marked in one of the subunits of the spike protein that is marked in blue, and ACE2 proteins are marked in green. (**C**) The infection of Huh-7 cells by 95 pseudotyped viruses. The data were displayed as mean ± SD (n = 3). The red dot line indicates the two-fold threshold compared to transduction rates of the WT proteins, while the blue dot line indicates the 50% threshold compared to transduction rates of the WT proteins.

The spike protein, which serves as the attachment factor on the surface of SARS-CoV-2, plays a pivotal role in viral entry and cross-species transmission. To screen all the potential mutations with higher possibilities, our initial step involved the retrieval of 2.9 million spike genes from the GISAID database, and calculating the mutation rates across the spike proteins (Figure 4A). These analyses uncovered 115 sites exhibiting a nonsynonymous mutation rate of ≥0.1%. Notably, of these sites, 75 sites were situated in the S1 region—nearly double the number identified within the S2 region (40 sites, Supplementary Figure S3). This trend was consistent at higher thresholds of mutation rates, specifically ≥0.2% and ≥0.3% (Supplementary Figure S3). These observations are in line with prior studies, which suggest that mutations in the S1 region are associated with aspects of enhanced fitness [1].

Referring to the abundance of genome sequences available in the GISAID database, 52 sites in the spike gene were selected for further research, as mutations occurring at these sites with higher mutation rate confer a degree of mutational tolerance. In detail, 13 sites situated in the receptor binding domain (RBD) of the S protein displayed higher mutation rates with mutation rates ≥0.1%; the remaining 39 sites were located in other regions of the protein that showed even higher mutation rates, at or exceeding 0.3% (Figure 4A,B). It was observed that mutations in those sites, such as, S373P, L452R, S477N, T478K, and G496S were associated with higher infectivity in human beings (Figure 4B) [13,14], while T95I, T572I, and P681H were demonstrated to enhance viral entry in pseudotyped assays involving cell cultures derived from tree shrews, pigs, and bats, respectively (Figure 4B) [18]. Subsequently, leveraging the principle of Codon Degeneracy, all the possible mutations at these 52 sites were predicted in silico, resulting in 191 possible non-synonymous mutations in reference to the wuhan-1 S protein. Of them, 96 mutations had already been recorded (before 25 July 2022), while the remaining 95 possible single-site non-synonymous mutations are yet to be observed (Figure 4B). Among the 95 prospective mutations, 67 were located in the S1 segments of the S protein, with an additional 22 in the RBD—a known hotspot for virus adaptive evolution. Thereafter, to explore their infection effects, we engineered 95 enhanced green fluorescent protein (EGFP)-encoded pseudotyped viruses, each bearing one of these mutated spike proteins, and referred to them collectively as VSVΔG-SARS2-S_mut_.

Because human populations may still represent the main reservoir for SARS-CoV-2, our primary objective was to test the impact of 95 mutations on viral entry using human cells, specifically employing the human hepatoma Huh-7 cell line, resulting in an overall transduction rate ranging from 2.5–22.0% (Figure 4C, Supplementary Table S4). Compared to wildtype S protein, most of these mutations have no significant impact on the infectivity of VSVΔG-SARS2. However, 13 notable mutations at 10 distinct sites significantly enhanced the transduction rates of VSVΔG-SARS2-S_mut_ pseudotyped viruses by over 2-fold, compared to that of the wildtype S protein (6.3%) (Supplementary Table S4). These impactful mutations included L24C (14.5%), R158M (16.0%), R158N (13.8%), L212Y (22.0%), S373C (13.3%), S373W (20.4%), S375W (17.6%), T376M (17.4%), T376R (16.4%), R408M (17.1%), G446E (14.6%), L452H (13.9%), and Y505C (14.8%). Conversely, mutations like L212W (2.4%) and Q613P (2.9%) were found to decrease the transduction rates of VSVΔG-SARS2-S_mut_ (Figure 4C). Notably, all 10 of these sites were found within the S1 segment of the spike protein, encompassing three sites in NTD and seven sites in the RBD domain. This not only demonstrates the potential of RBD mutations to influence the S-ACE2 interaction, but also underscores the significant role played by mutations outside of the RBD domain in influencing viral entry [18].

We further observed mutations at site R158G, which is a feature of the B.1.617.2 (Delta) variant and affects the steric clash with other residues [34,35]. While the dominance of the Delta variant dwindled with the rise of the Omicron variants, leading to a decrease in R158 mutations, we noted that the mutations rates increased at the end of 2023 (Supplementary Figure S4). Mutations at L24, S373, S375, T376, R408, G446, L452, and Y505 are hallmark molecular features of SARS-CoV-2 Omicron variants (Supplementary Table S5) [36]. Coinciding with the circulation of Omicron variants, mutations in these sites accumulated rapidly during 2022 and 2023 (Supplementary Figure S4) [37]. We also performed molecular dynamic simulations of hACE2 with WT spike and 13 mutated spikes using AMBER 20 [27]. The results showed that the binding free energy of hACE2 and spike proteins range from −32.4 to −52.3 KJ/mol, with S373W showing the greatest binding affinity (Supplementary Figure S5). From the result, we also observed that the absolute value of the binding free energy of L452H-hACE2 and Y505C-hACE2 are slightly lower than WT spike-hACE2; we speculate that the interaction of spike with other receptors or auxiliary receptors might contribute to better viral entry [38]. Whether mutations in these sites contribute to heightened transmissibility individually or in synergy remains to be clarified; however, our findings indicate that potential mutations at these sites do indeed facilitate enhanced spike-mediated viral entry. Consequently, this underscores the importance of vigilant monitoring for mutations at these specific genomic locations.

### 3.4. Infection of Susceptible Animals by Pseudotyped Viruses Bearing Mutated S Proteins

**Figure 5:**
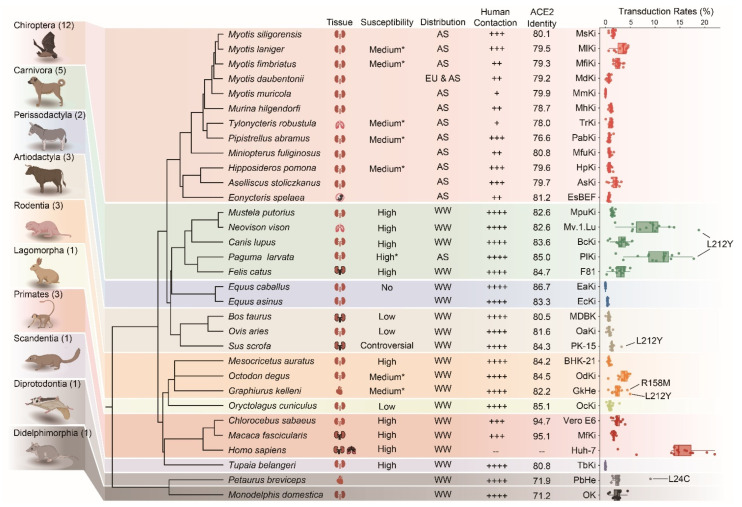
Infection animal cell cultures by different pseudotyped viruses bearing mutated S protein. Tested species were displayed as their position in the phylogenetic tree (TimeTree, https://timetree.org/home (accessed on 9 October 2024)). 34 cell cultures from 31 species were derived from the kidneys, heart, lungs, and other tissues. These species displayed variant susceptibility based on previous research. Cells were grouped by their distribution, their human–animal contacts, and their sensitivity to different pseudotyped viruses. High represents the species that are highly susceptible to SARS-CoV-2 infection; medium represents the species that can be occasionally infected by SARS-CoV-2; low represents the species are rarely infected by SARS-CoV-2, the asterisk (*) represents the species are assessed by in vitro infection analysis. +, ++, +++, and ++++ represent rarely-, occasionally-, medium-, and frequently human-animal contact.

Since these 13 mutations of SARS-CoV-2 show significantly enhanced transduction rates to human cells, we further measured their impact on cross-species transmission using cell cultures that derived from a range of domestic, companion, and wild animal species. In total, 31 species from ten orders were selected, including Diprotodontia (one species), Didelphimorphia (one species), Scandentia (one species), Primates (three species), Lagomorpha (one species), Rodentia (three species), Perissodactyla (two species), Artiodactyla (three species), Carnivora (five species), and Chiroptera (12 species) (Figure 5). Among these tested species, 13 species had been verified by natural or experimental infection. Additionally, eight species were previously predicted to be vulnerable based on in vitro infections from our earlier studies (Figure 5) [18].

Based on the results, cell cultures from humans, palmed civets, minks, and Chinese water myotis are more sensitive to VSVΔG*-SARS2-S_mut_ pseudotyped viruses (Figure 5, Supplementary Table S6). Specifically, S373P and L452R were found to enhance the binding affinity between spike and hACE2. However, spike with mutations S373C, S373W, and L452H did not demonstrate an increased capacity to facilitate viral entry into cell cultures from other animal species. Interestingly, we noted that spike protein L24C mutation significantly enhances the transduction rates of the VSVΔG*-SARS2-S_mut_ virus to sugar glider heart (PbHe) cells with 9.1% of cells being transduced, compared to 2.5% of cells been transduced by VSVΔG*-SARS2 viruses. Moreover, the R158M mutation substantially improved the transduction efficiency of VSVΔG*-SARS2-S_mut_ to Kellen’s dormouse heart cells (GkHe), with a transduction rate of 4.5% of cells, representing approximately a 3.3-fold increase over the wildtype spike protein. Additionally, L212Y in spike protein confer VSVΔG*-SARS2-S_mut_ a superior ability to infect mink cells (Mv.1.Lu, 18.8% vs. 5.5%), palmed civet cells (PlKi, 17.8% vs. 6.5%), and Kellen’s dormouse heart cells (GkHe, 5.0% vs. 1.4%).

The frequency and nature of contact between humans and animal species are critical factors in the likelihood of successful spillover events. To refine our understanding of this dynamic, we assessed the potential for contact between the species tested and with humans. Among these species, domestic and companion animals have notably close interactions with human beings. For instance, Kellen’s dormouse, commonly kept as pet, exhibited increased sensitivity to the pseudotyped virus sporting the L24C and L212Y mutations in the spike protein. Similarly, palmed civets, which are utilized as a meat source, are more sensitive to L212Y-harboring pseudotyped virus. Bats are recognized as significant reservoir hosts for numerous impactful zoonotic pathogens. In this study, we evaluated 12 bat species commonly found across Asia. Encouragingly, among these bat species, we did not detect any particular mutations that conferred upon SARS-CoV-2 an improved capacity to infect bat cells (Figure 5). This outcome emphasizes the necessity for the rigorous monitoring of domestic and companion animals, alongside certain bat species, in order to surveil and mitigate potential spillover events involving SARS-CoV-2.

## 4. Conclusions and Discussion

The bidirectional transmission of SARS-CoV-2 has been a significant concern since the inception of the pandemic. Here, we examined the plausible host range of SARS-CoV-2 Delta and Omicron variants. Our results suggested that the host range of SARS-CoV-2 wuhan-hu-1 strain, B.1.617.2 (Delta), XBB, XBB.1.5, XBB.1.16, EG.5.1, BA.2.75, and BA.2.86 variants shared similar host tropism with minor changes. We also tested the host range of two SARSr-CoVs, the results showed that RaTG13 and RmYN02 had the ability to potentially infect a broad range of mammalian animals. Furthermore, we predicted the potential future mutations of the spike (S) protein and identified 13 potential mutations that could enhance the infectivity of SARS-CoV-2 to human cells. Further evidence showed that mutations L24C, R158M, and L212Y significantly promote the infection of SARS-CoV-2 to several animal species. This finding serves as a crucial reminder of the need for vigilance surrounding these particular mutations during future monitoring and surveillance efforts for SARS-CoV-2 in both human and animal populations.

The SARS-CoV-2 virus continues to undergo evolutionary changes. Throughout the course of this research project, novel dominant omicron variants of the SARS-CoV-2 virus have emerged repeatedly. Based on our findings, we hypothesize that different variants of SARS-CoV-2 share a similar host range; however, there is potential for expansion into new hosts. For instance, white-tailed deer are certainly not the sole species harboring SARS-CoV-2 during its circulation. Hence, ongoing and systematic monitoring of SARS-CoV-2 in animal populations should be implemented. Moreover, molecular dynamic simulations showed that these mutations might impact the binding affinity of S-ACE2 by affecting the interaction of several key animo acids; however, further research is necessary to elucidate the precise mechanisms involved. It is also important to acknowledge the variability in infection dynamics between the use of pseudotyped viruses in experiments and actual viral infections. While pseudotyped viruses can inform us about spike protein-mediated viral entry specifically, it is crucial to consider that other viral components, such as the RNA polymerase, may also play key roles in crossing species barriers. Nonetheless, successful viral entry is a fundamental precursor to subsequent steps of infection. The analysis of pseudotyped virus infection provides crucial insights into the host range of SARS-CoV-2. However, it is important to acknowledge that these cellular-level infection analyses may not fully reflect real-world infections. In summary, as SARS-CoV-2 continues to evolve, we must remain cautious and alert to the potential future risks it may pose.

## Data Availability

The original contributions presented in the study are included in the article/Supplementary Materials, further inquiries can be directed to the corresponding author.

## Supplementary Materials

The following supporting information can be downloaded at https://www.mdpi.com/article/10.3390/genes15101321/s1, Figure S1. VSV pseudotyped viruses bearing S proteins from different SARS-CoV-2 variants could transduce cell cultures from a broad range of mammalian species. (A) The overall transduction of VSV pseudotyped viruses bearing different SARS-CoV-2 S proteins was displayed in violin plots. VSV∆G*-SARS2-XBB.1.16 and VSV∆G*-SARS2-BA.2.86 exhibited a significantly higher capacity to transduce the tested cell cultures, with *p*-values of <0.01 (**) and <0.001(***), respectively. The boxplot embedded within the violin plot illustrates the quantiles of overall transduction rates. Statistical analyses were conducted using a paired Wilcoxon test. (B) Human Huh-7 cell line could be transduced by different pseudotyped viruses without significant differences. (C) VSV∆G*-SARS2-B.1.617.2, VSV∆G*-SARS2-XBB, VSV∆G*-SARS2-XBB.1.5, VSV∆G*-SARS2-XBB.1.16, and VSV∆G*-SARS2-BA.2.86 showed higher capacity to transduce primary cell cultures from Asian particolored bat (AsKi). (D) VSV∆G*-SARS2-B.1.617.2 showed higher efficiency to transduce primary cell culture from dog (BcLu), while VSV∆G*-SARS2-XBB, VSV∆G*-SARS2-XBB.1.5, VSV∆G*-SARS2-XBB.1.16, and VSV∆G*-SARS2-EG.5.1 displayed decreased capacity to transduce cell cultures from dog (BcLu). Figure S2. VSV pseudotyped viruses bearing S protein from RaTG13 and RmYN02 could also transduce cell cultures from a broad range of mammalian species. VSVΔG*-RaTG13 showed higher capacity to transduce cell cultures than VSVΔG*-RmYN02, either in total cell cultures (A, *p* < 0.05) or in bat cell cultures (B, *p* < 0.05). The boxplots embedded within the violin plot illustrate the quantiles of overall transduction rates. Statistical analyses were conducted using a paired Wilcoxon test. Figure S3. Comparison of number of mutations in S1 and S2 segments. The results showed that mutations in S1 segment is more frequent than that in S2 region. Figure S4. The amino acid substitution rates across time. The majority of selected mutations accumulated along with the circulation of omicron variants of SARS-CoV-2. Figure S5. Molecular dynamic simulation of the binding affinity between mutated S proteins and hACE2 receptor. Table S1. Details of tested animals. Table S2. Comparison of different tested S proteins. Table S3. Transduction rates of pseudotyped viruses bearing spike proteins from novel variants and Bat-CoVs. Table S4. Transduction rates of Huh-7.5 cell lines by pseudotyped viruses bearing spike proteins with potential mutations. Table S5. Sites with high frequency mutation rates are important molecular signatures of many SARS-CoV-2 variants. Table S6. Transduction rates of animal cell cultures by pseudotyped viruses bearing spike proteins with potential mutations.
