# Supplementary material for: Cross-Species Susceptibility of Emerging Variants of SARS-CoV-2 Spike
Source: Genes (Basel). 2024 Oct 14;15(10):1321. doi: 10.3390/genes15101321 (PMC11507407; doi:10.3390/genes15101321)
Supplement: Supplementary file 1 [file genes-15-01321-s001.zip › Supplementary Figs.pdf]

Supplementary information for

Cross-Species Susceptibility of Emerging Variants of SARS-CoV-2 Spike

Meng Li[1], Fei Lv[1], Zihao Li[1,2], Chenyu Zhao[1,2], Xiao Wang [1], Pingfen Zhu[1], Xuming Zhou[1*]

[1]Key Laboratory of Animal Ecology and Conservation Biology, Institute of Zoology, Chinese Academy of Sciences, Beijing 100101, China

[2]University of Chinese Academy of Sciences, Beijing 100049, China

*Correspondence: zhouxuming@ioz.ac.cn.

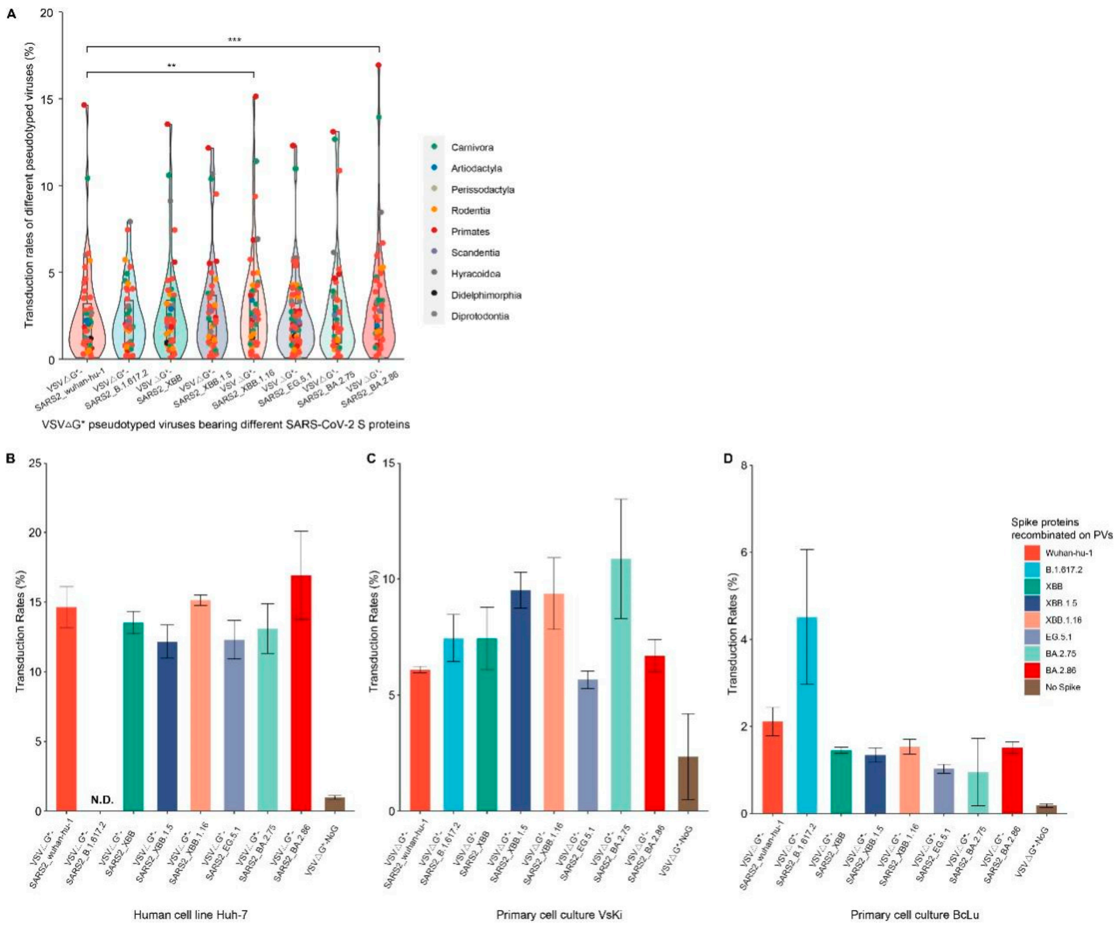

Supplementary Figure S1. VSV pseudotyped viruses bearing S proteins from different SARS-CoV-2 variants could transduce cell cultures from a broad range of mammalian species. (**A**) The overall transduction of VSV pseudotyped viruses bearing different SARS-CoV-2 S proteins was displayed in violin plots. VSVΔG*-SARS2-XBB.1.16 and VSVΔG*-SARS2-BA.2.86 exhibited a significantly higher capacity to transduce the tested cell cultures, with P-values of < 0.01 (**) and < 0.001(***), respectively. The boxplot embedded within the violin plot illustrates the quantiles of overall transduction rates. Statistical analyses were conducted using a paired Wilcoxon test. (**B**) Human Huh-7 cell line could be transduced by different pseudotyped viruses without significant differences. (**C**) VSVΔG*-SARS2-B.1.617.2, VSVΔG*-SARS2-XBB, VSVΔG*-SARS2-XBB.1.5, VSVΔG*-SARS2-XBB.1.16, and VSVΔG*-SARS2-BA.2.86 showed higher capacity to transduce primary cell cultures from Asian particolored bat (AsKi). (**D**) VSVΔG*-SARS2-B.1.617.2 showed higher efficiency to transduce primary cell culture from dog

(BcLu), while VSVΔG*-SARS2-XBB, VSVΔG*-SARS2-XBB.1.5, VSVΔG*-SARS2-XBB.1.16, and VSVΔG*-SARS2-EG.5.1 displayed decreased capacity to transduce cell cultures from dog (BcLu).

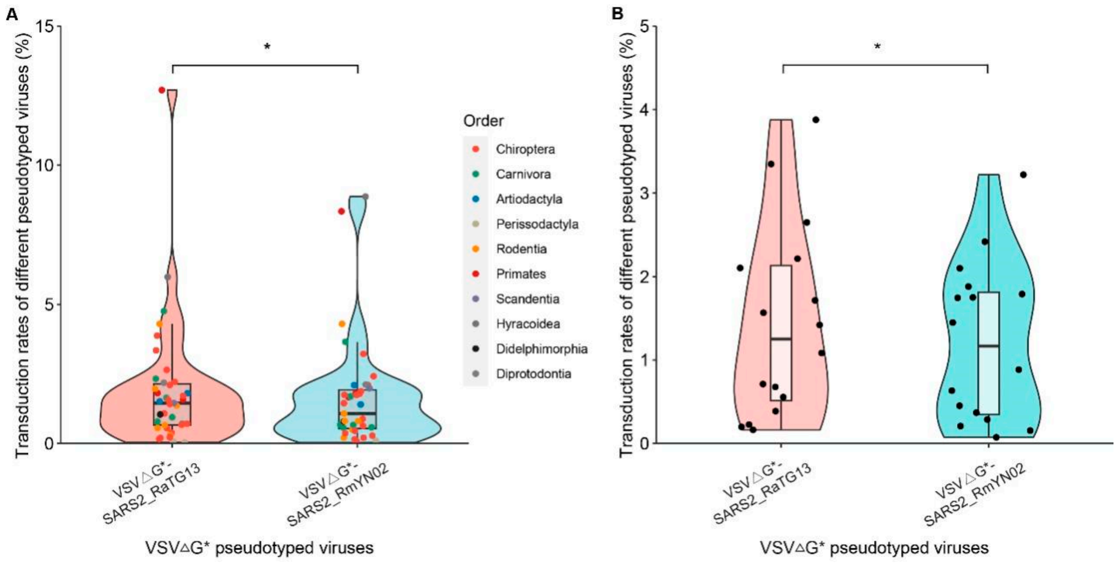

Supplementary Figure S2. VSV pseudotyped viruses bearing S protein from RaTG13 and RmYN02 could also transduce cell cultures from a broad range of mammalian species. VSVΔG*-RaTG13 showed higher capacity to transduce cell cultures than VSVΔG*-RmYN02, either in total cell cultures (**A,** $P < 0.05$) or in bat cell cultures (**B,** $P < 0.05$). The boxplots embedded within the violin plot illustrate the quantiles of overall transduction rates. Statistical analyses were conducted using a paired Wilcoxon test.

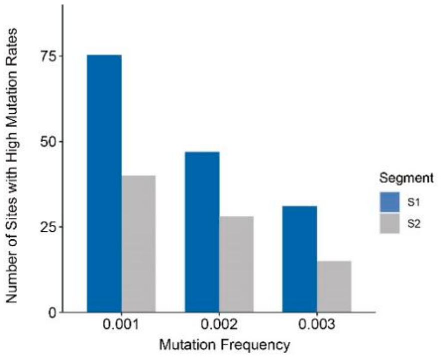

Supplementary Figure S3. Comparison of number of mutations in S1 and S2 segments. The results showed that mutations in S1 segment is more frequent than that in S2 region.

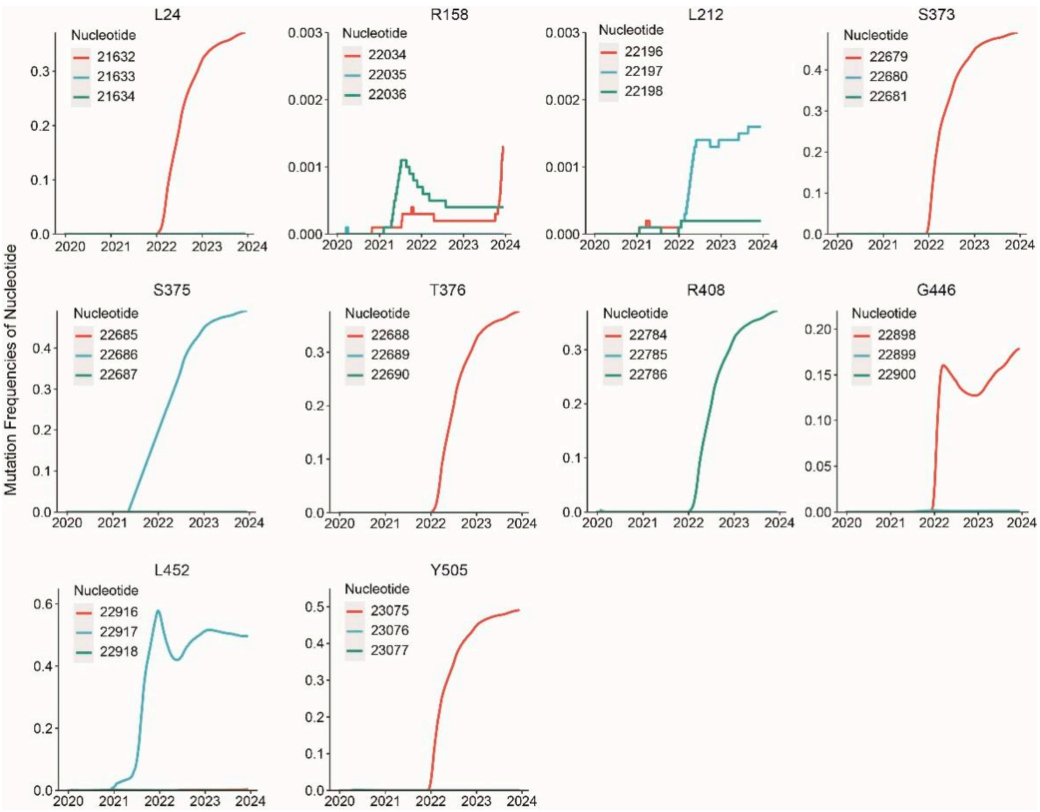

Supplementary Figure S4. The amino acid substitution rates across time. The majority of selected mutations accumulated along with the circulation of omicron variants of SARS-CoV-2.

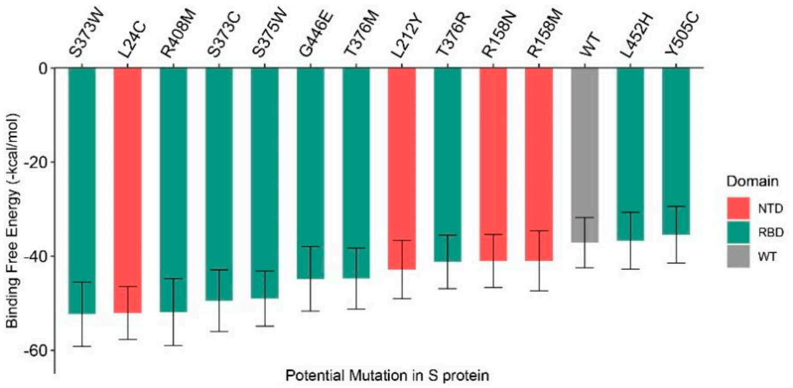

Supplementary Figure S5: Molecular dynamic simulation of the binding affinity between mutated S proteins and hACE2 receptor.
